# Prenatal diagnosis of a rare *β*‐thalassemia gene －90 (C>T) (*HBB*: c.‐140 C>T) mutation associated with deletional Hb H disease (‐‐^SEA^/‐*α* ^4.2^)

**DOI:** 10.1002/mgg3.1472

**Published:** 2020-09-03

**Authors:** Hou Qian, Jianlin Huang, Ji Xu, Weihua Zhao, Xiufeng Ye, Wenlan Liu

**Affiliations:** ^1^ The Medical Genetics & Molecular Diagnosis Laboratory Shenzhen China; ^2^ Prenatal Diagnosis Center Shenzhen China; ^3^ Department of Obstetrics Shenzhen Second People's Hospital/the First Affiliated Hospital of Shenzhen University Health Science Center Shenzhen China

**Keywords:** Hb H disease, prenatal diagnosis, rare mutation, thalassemia

## Abstract

**Background:**

Hemoglobin H (Hb H) disease can be caused by compound heterozygosity for two different mutations or from homozygotes for mutations, and conventional genetic methods may lead to misdiagnosis when Hb H disease is combined with a rare β‐thalassemia.

**Methods:**

Hematology parameters and hemoglobin electrophoresis analysis, gap‐polymerase chain reaction (gap‐PCR) and reverse dot‐blot hybridization (RDB‐PCR) were employed to identify common α‐thalassemia and Hb H disease. Rare β‐thalassemia mutations were detected by DNA sequencing.

**Results:**

Hematological analysis and hemoglobin electrophoresis revealed a mild anemia α^0^‐thalassemia trait (Hb 90 g/L, MCV 71 fL, and MCH 22.7 pg) compound with *β* ^+^‐thalassemia trait (MCV 71 fL, MCH 22.7 pg, and HbA2 5.51%) for the pregnant woman. DNA sequencing for the β‐globin gene revealed rare a －90 (C>T) (*HBB*: c.‐140 C>T) mutation for the woman. DNA analysis identified that the fetus inherited the α^0^‐thalassemia mutation [‐‐^SEA^ (Southeast Asian)] and a rare *β* ^+^‐thalassemia mutation －90 (C>T) (*HBB*: c.‐140 C>T) from the mother, and the *α* ^+^‐thalassemia mutation [‐*α* ^4.2^ (leftward)] from the father.

**Conclusion:**

We reported a rare －90 (C>T) (*HBB*: c.‐140 C>T) mutation combined with the ‐‐^SEA^/‐*α* ^4.2^ in a family. This finding enriched the mutation spectrum of thalassemia molecular characteristics in China and emphasized the significance in DNA sequencing in mutation screening for the families with thalassemia.

## 1 INTRODUCTION

Thalassemia is a group of hereditary hematopathy caused by globin chains synthesis disorder (Martin & Thompson, 2013), which is common in the tropics and subtropics. In China, Guangdong, Guangxi and Hainan are the high incidence areas (He et al., 2017; Zhao, Weng, & Wu, 2018). The pathogenic cause of α‐thalassemia contains deletions or mutations that eliminate one or both α‐globin genes (*HBA1* and *HBA2*; OMIM: 141800 and 141850) from the influenced chromosome 16 (Zeng & Huang, 1985), and can be subdivided into α^0^‐thalassemia (the expression of two α‐globin genes on one allele is absent) and α^+^‐thalassemia (the expression of a single α‐globin gene is reduced or absent). In general, heterozygosity status of α^+^‐thalassemia or α^0^‐thalassemia is clinically asymptomatic, whereas the complex heterozygosity status of α^+^‐thalassemia and α^0^‐thalassemia is Hb H disease. Hb H disease is the most common α‐thalassemia with intermediate severity. Genetically, Hb H disease is caused by interaction of α^0^‐ thalassemia and α^+^‐thalassemia, which can be divided into a deletional form (deletion of three α‐globin genes) and non‐deletional form (deletion of two α‐globin genes compound a non‐deletional mutation), leaving only one intact α‐globin gene. The disease with α^0^‐ thalassemia and a point mutation on the α2‐globin gene is more serious, particularly in individuals with the combinations of α^0^‐ thalassemia and super unstable α‐globin variants (Chui, Fucharoen, & Chan, 2003; Weatherall, 2001). β‐thalassemia is characterized by the decreased (β^+^) or no‐intact synthesis (β^0^) of *β*‐globin chains due to mutations on the *β*‐globin gene (*HBB*; OMIM: 141900) located on the chromosome 11 (Galanello & Origa, 2010), leading to the imbalance of the ratio between *α*‐ and *β*‐globin chain (Sankaran & Nathan, 2010; Weatherall, 2010). Carriers of β^0^ and β^+^ thalassemia alleles have some hematological phenotypes, such as mild anemia, microcell low pigment red blood cell index, elevated HbA_2_, or slightly elevated Hb F levels (Thein, 2018). Currently, more than 900 mutations of *HBB* have been listed in human globin database (HbVar: a database of human hemoglobin variants and thalassemias, 2016). More than 30 diverse mutations of *β*‐thalassemia have been detected in Chinese populations (Xie, Zhou, & Xiao, 2016). The −90 (C>T) (*HBB*: c.‐140 C>T) is a rare C>T mutation at nucleotide −90 within the CACCC motif of the β‐globin gene promoter (Prajantasen, Teawtrakul, Fucharoen, & Fucharoen, 2014), which has been previously reported in Portuguese, Chinese, Indian, Pakistani, and Thai, respectively (Faustino et al., 1992; Gorakshakar, Nadkarni, Phanasgaonkar, Colah, & Mohanty, 2005; Jia et al., 2003; Moatter, Kausar, Aban, Ghani, & Pal, 2012; Prajantasen et al., 2014). The β‐globin mutations can affect mRNA transcription and globin chain stability (Myers, Tilly, & Maniatis, 1986). Since thalassemia is widespread in south of China, there are a large number of carriers for the same genotypes of thalassemia that may give birth to fetuses with severe thalassemia. Therefore, the pregnant women in this area are often encouraged to assess gene mutations for thalassemia during pregnancy, and indeed prenatal diagnosis of thalassemia has been approved to significantly reduce the births of children with severe thalassemia.

In this paper, we reported a 23‐year‐old pregnant woman who showed a typical α^0^‐thalassemia (‐‐^SEA^ deletion) trait (Hb 90 g/L, MCV 71 fL, MCH 22.7 pg) and a *β* ^+^‐thalassemia －90 (C>T) (*HBB*: c.‐140 C>T) phenotype (MCV 71 fL, MCH 22.7 pg and HbA2 5.51%) and her fetus was identified to have rare compound heterozygotic mutations of the ‐‐^SEA^/‐*α* ^4.2^ deletions combined with *β* ^+^‐thalassemia gene －90 (C>T) (*HBB*: c.‐140 C>T) mutation (Myers et al., 1986), which was inherited from the pregnant mother and the *α*‐thalassemia carrier father *α* ^+^‐thalassemia mutation [‐*α* ^4.2^ (leftward)]. This report enriched the mutation spectrum of thalassemia molecular characteristics in China and could help with the prenatal diagnosis of thalassemia.

## 2 METHODS

### 2.1 Ethical statement

This study was approved by the Ethics Committee of Shenzhen Second People's Hospital (Approval No: 20200422009) and written informed consents were obtained from the couple.

### 2.2 Patients

A 23‐year‐old pregnant wife and her husband from Guangdong Province, Southern China came to our prenatal diagnosis center for genetic counseling at the 13th week of pregnancy because they were both *α*‐thalassemia carriers. Amniocentesis was performed for prenatal diagnosis at the 19th week of pregnancy.

### 2.3 Analysis of hematological parameters and hemoglobin components

The hematological parameters were interpreted with a Sysmex XN‐1000 automated blood cell counter (Sysmex Co. Ltd., Kobe, Japan). The hemoglobin components were detected by hemoglobin electrophoresis instrument (V8, Helena, Beaumout, USA).

### 2.4 DNA sample preparation

The couple's genomic DNA was extracted with an automatic nucleic acid extractor at Kaishuo Biological Co., Ltd (Xiamen, China). The genomic DNA of fetal amniotic fluid exfoliated cells was extracted using QIAamp DNA Mini Kit (Dusseldorf, Germany).

### 2.5 Gene analysis

The gene deletion mutation analysis of *α*‐thalassemia 1 (‐‐^SEA^ and ‐‐^THAI^ deletions) and *α*‐thalassemia 2 (‐*α* ^3.7^ and ‐*α* ^4.2^ deletions) was performed using a thalassemia genotyping kit. The 17 common *β*‐thalassemia mutations including CD41‐42 (‐TCTT), CD43 (G‐T), −28 (A‐G), −29 (A‐G), CD 27/28 (+C), −30 (T‐C), −32 (C‐A), CD71/72 (+A), CD26 (G‐A),CD17 (A‐T), CD31 (‐C), CD14/15 (+G), IVS‐I‐1 (G‐T), IVS‐I‐5 (G‐C), IVS‐II‐654 (C‐T), Cap+1 (A‐C), and initiation codon (ATG‐AGG) were assessed using the PCR combined with reverse dot hybridization technique (PCR‐RDB). The non‐deletion mutation *α*‐thalassemia (Hb CS, Hb QS and Hb Westmead) were diag nosed using PCR‐RDB. All the above reagents were purchased from Yilifang Biological Technology Company (Shenzhen, China).

### 2.6 DNA sequencing

The *HBB* gene (GenBank accession number: NG_000007.3) was explored by sequencing analysis of three amplified fragments as shown in Table 1. The amplification was performed on PCR machine (C‐1000, Bio‐Rad, Hercules, USA) using 100 ng of genomic DNA, 10 pmol of forward and reverse primers. The 25 μL PCR reaction mixture contained 12.5 μL of Premix PrimeSTAR HS (Baobio Engineering Dalian Co., Ltd, Dalian, China), 1.5 μL of 10 pmol/L forward and reverse primers, 100 ng of genomic DNA. After initial heating at 95 °C for 5 minutes, a total of 32 PCR cycles were performed under the following PCR conditions: 97 °C for 45 seconds, 66 °C for 30 seconds, and 72 °C for 180 seconds and a final extension at 72 °C for 10 minutes. The PCR products were sequenced on an ABI PRISMTM 3130 xl automated sequencer (Applied BioSystems, Walsham, USA).

**TABLE 1:** The sequencing primers of *β*‐globin gene.

Names	Primers
*HBB*1 forward	AACTCCTAAGCCAGTGCCAGAAGAGC
*HBB*2 forward	GTGTACACATATTGACCAAA
*HBB*3 reverse	ATGCACTGACCTCCCACATTCCC

## 3 RESULTS

### 3.1 Hematological indexes and Hb analysis

**FIGURE 1:**
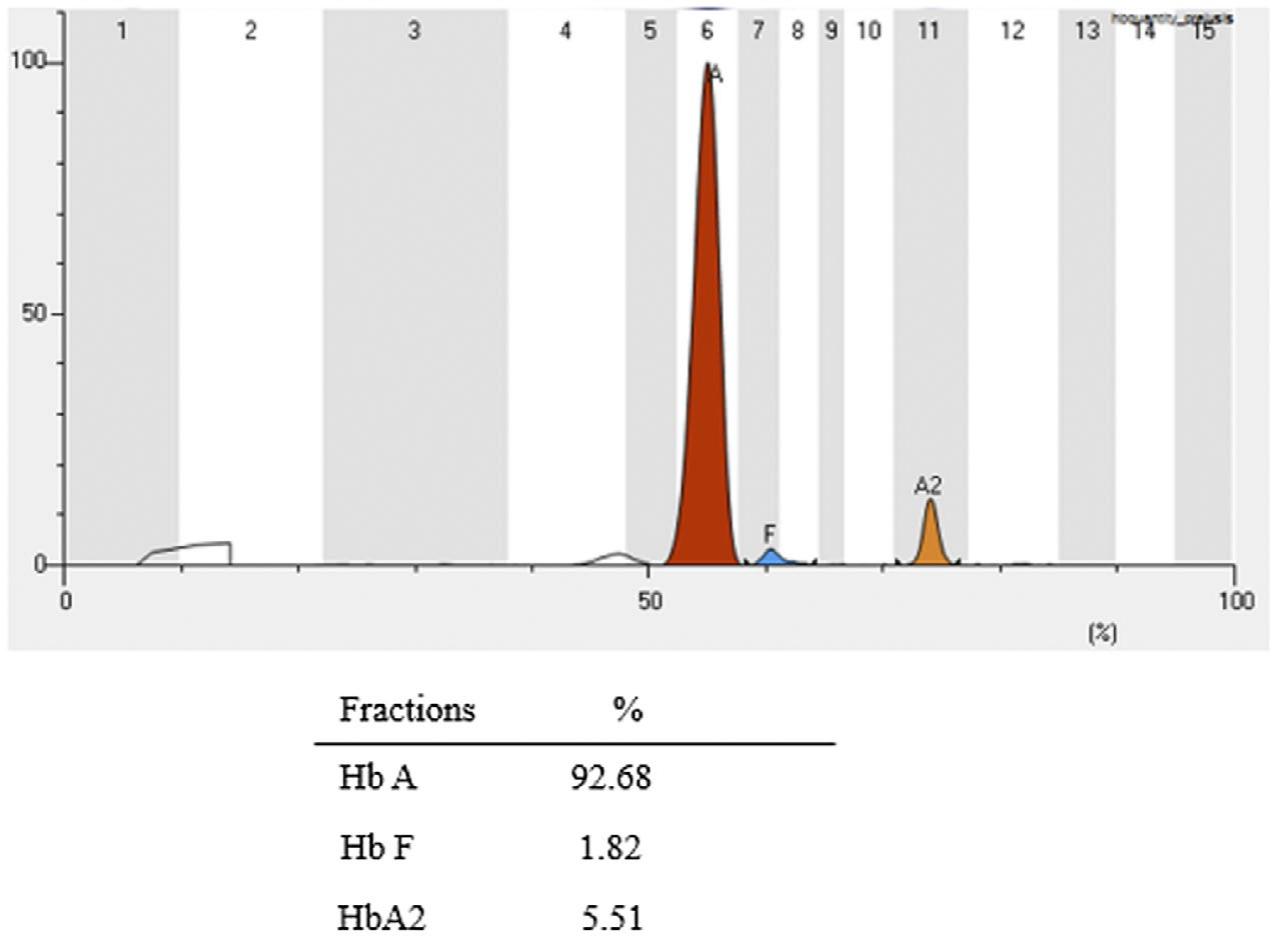
Result of the pregnant women by hemoglobin electrophoresis. By Helena V8, the results of the three most common hemoglobin bands and fractions are displayed using the software Platinum program. Peak of the Hb A, Hb F, and Hb A2 appears in a specific zone: Hb A (92.68%) =6, Hb F (1.82%) =7 and Hb A_2_ (5.51%) =11. According to the results analysis showed a higher peak with Hb A2 for the pregnant women.

Hematological indices of the pregnant wife are listed in Table 2, with, 90 g/L Hb (normal range: 120‐160 g/L),71.0 fL mean corpuscular volume (MCV) (normal range: 80.0‐100.0 fL), and 22.7 pg mean corpuscular hemoglobin (MCH) (normal range: 27.0‐34 pg), supporting the diagnosis of mild microcytic hypochromic anemia. Hb electrophoresis showed an increased peak with Hemoglobin A_2_ (HbA_2_) of 5.51% (normal range: 2.5‐3.5%) for the pregnant wife (Figure 1). Of note, the husband showed normal hematological indices and normal HbA_2_ value (Table 2).

**TABLE 2:** Hematological data and globin genotype of patients.

Parameters	pregnant woman	Husband	Fetus
Age (years)	23	25	18 (week)
Hb (g/L)	90	147	ND
MCV (fL)	71	85.5	ND
MCH (pg)	22.7	27.1	ND
Hb A (%)	92.68	97.40	ND
Hb F (%)	1.82	0	ND
Hb A_2_ (%)	5.51	2.60	ND
DNA (*α*‐genes)	‐‐^SEA^/*αα*	‐*α* ^4.2^/*αα*	‐‐^SEA^/‐*α* ^4.2^
DNA (*β*‐genes)	－90 (C>T) *HBB*: c.−140 C>T	β^N^ /β^N^	－90 (C>T) *HBB*: c.−140 C>T

Abbreviation: ND, no detection.

### 3.2 Gene analysis of thalassemia by gap‐PCR and DNA sequencing

**FIGURE 2:**
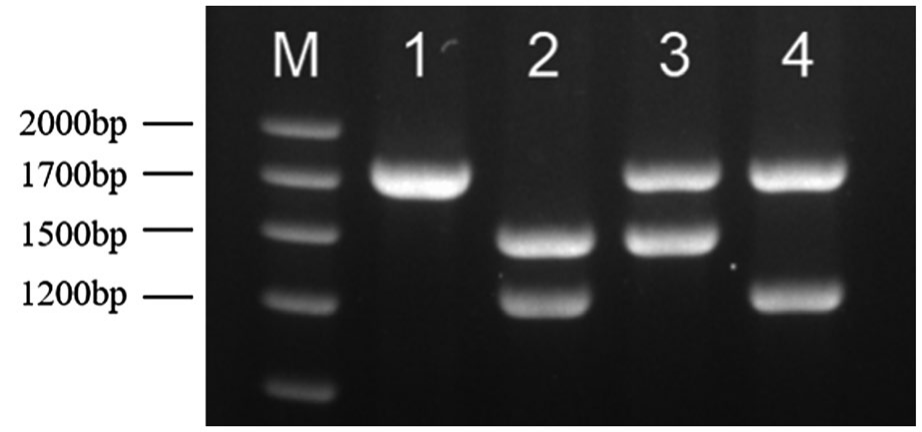
Detection results of *α*‐globin gene deletion by gap‐PCR. M = Marker 1700 bp. Lanes 1–4 were the normal sample, fetus, husband, and pregnant woman. Lane 1 was normal sample and the genotypes were αα/αα. Lane 2 was fetus sample and the genotypes were ‐‐^SEA^/‐*α* ^4.2^. Lane 3 was the husband and the genotypes were ‐*α* ^4.2^/*αα*. Lane 4 was the pregnant woman and the genotypes were ‐‐^SEA^ /*αα*.

**FIGURE 3:**
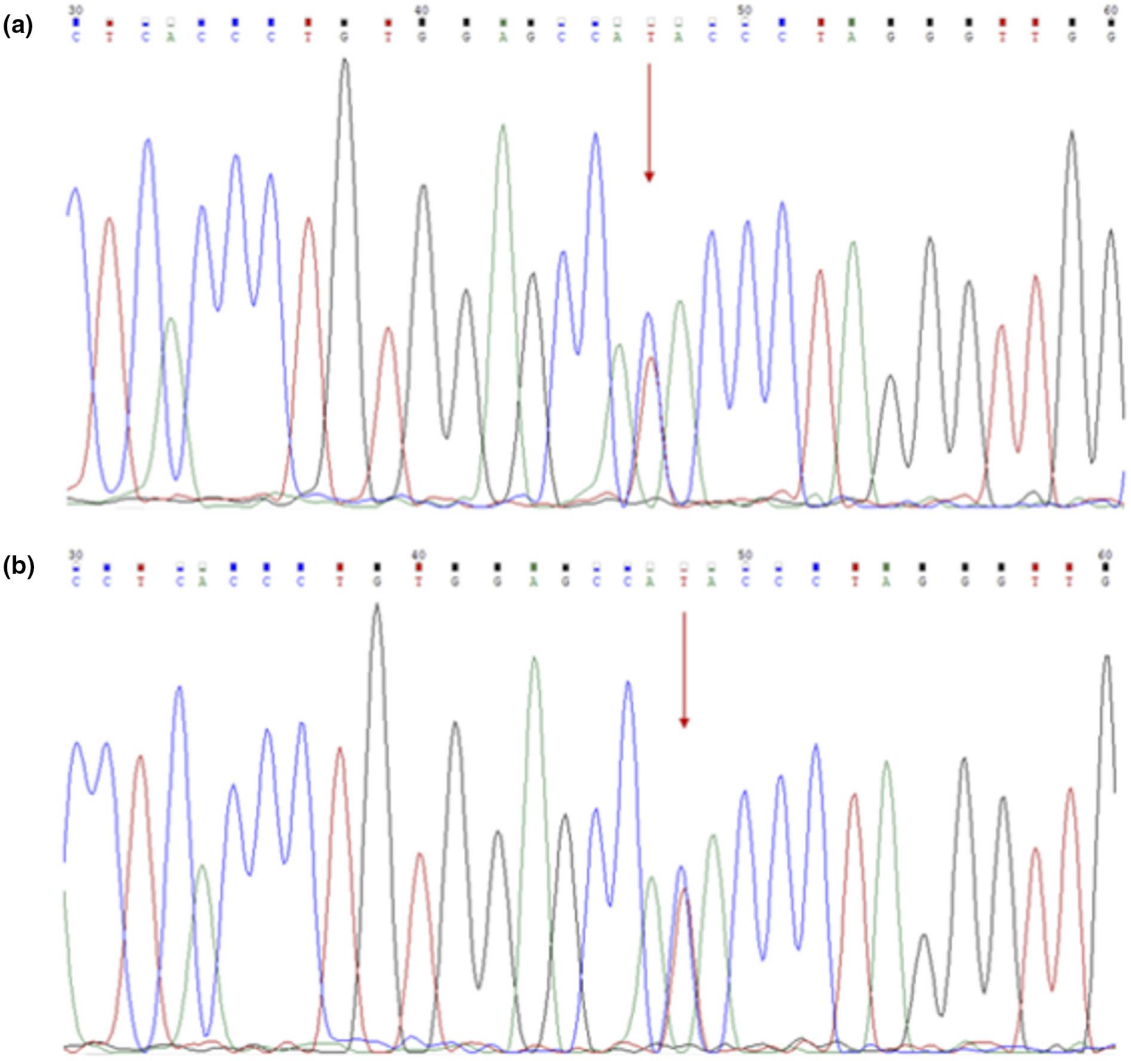
Sequence analysis of the amplified *β*‐globin gene. The arrow indicates the －90 (C>T) (*HBB*: c.‐140 C>T). A: Forward sequencing result of the pregnant woman showing the mutation of －90 (C>T) (*HBB*: c.‐140 C>T); B: Forward sequencing result of the fetus showing the mutation of －90 (C>T) (*HBB*: c.‐140 C>T).

The pregnant wife carried a ‐‐^SEA^ deletion as detected by gap‐polymerase chain reaction (gap‐PCR) (Figure 2). However, hematological examination showed a typical *β*‐thalassemia trait (MCV 71 fL, MCH 22.7 pg, Hb 90 g/d and HbA_2_ 5.51%). Therefore, we analyzed the seventeen common *β*‐thalassemia mutations using PCR‐RDB, but did not identify any known mutations. In this context, we speculated that a rare *β*‐thalassemia mutation might exist, and conducted DNA sequencing using Sanger dideoxy chain‐termination method. Indeed, we identified a rare *β*‐globin gene mutation －90 (C>T) (*HBB*: c.‐140 C>T) on the *β* gene, reflected by a double‐peak located at nt47 (Figure 3A). Gap‐PCR analysis showed that the husband carried the ‐*α* ^4.2^ deletion, which matched well with his hematological parameters and Hb analysis results (Figure 2). Apparently, the fetus co‐inherited both parental mutations and the genotype was ‐‐^SEA^/‐*α* ^4.2^ with －90 (C>T) (*HBB*: c.‐140 C>T) (Figure 3B). Taken together, these results showed that the husband was with a^−4.2^ *α*/*αα* genotype, the pregnant wife was with a ‐‐^SEA^/*αα* and －90 (C>T) (*HBB*: c.‐140 C>T) genotype, and the fetus was with a ‐‐^SEA^/‐*α* ^4.2^ and －90 (C>T) (*HBB*: c.‐140 C>T) genotype.

## 4 DISCUSSION

Thalassemia is a high incidence hereditary blood disease in southern China, especially for the major or intermediate thalassemia that can lead to death and disability, bringing a heavy burden to the family and the society. According to the current epidemiological survey data, the incidence of *α*‐thalassemia in Guangdong, Guangxi, Hainan, Taiwan, Hong Kong, and other regions is 4% ~15%, and *β*‐thalassemia is 1% ~6% (Xu et al., 2004). Thalassemia has grown up to be a public health problem in these areas. At present, prenatal screening and diagnosis of pregnant women have been the method of choice for avoiding the birth of children with severe thalassemia so as to achieve the goal of eugenics and childbirth (Barrett, Saminathan, & Choolani, 2017; Gilad et al., 2017; Rund, 2016). In this report, a co‐inheritance of rare *β*‐thalassemia point mutation with ‐‐^SEA^/‐*α* ^4.2^ was discovered after the blood routine test, hemoglobin electrophoresis analysis, gap‐PCR, and PCR‐RDB method followed by DNA sequencing. However, when usual methods are used to screen thalassemia genotypes, those rare types of mutations will be missed, resulting in missing diagnosis. Therefore, the discovery and detection of rare types of thalassemia should be so emphasized.

Here, we report for the first time a combination of －90 (C>T) (*HBB*: c.‐140 C>T) with the ‐‐^SEA^ and ‐*α* ^4.2^ deletion in the Chinese population. The hematological indices of the pregnant wife showed that erythrocyte MCV and MCH were low, and a common ‐‐^SEA^ deletion was found using gap‐PCR. Moreover, hemoglobin electrophoresis results showed that the pregnant wife had a classical *β*‐thalassemia trait (MCV 71 fL, MCH 22.7 pg and HbA_2_ 5.51%). DNA sequencing showed that she has a rare mutation of －90 (C>T) (*HBB*: c.‐140 C>T), a C>T transition within the proximal CACCC box of the β‐globin gene. The CACCC motif is an erythroid‐specific binding site of the erythroid Krüppel‐like factor (EKLF). The EKLF is a zinc‐finger transcription factor that plays critical roles in erythropoiesis including regulating β‐like globin gene switching. This －90 (C>T) mutation in the proximal CACCC/EKLF binding site disrupts the binding and trans‐activation of EKLF to the mutant promoter, leading to a 10‐fold decrease in the transcription of *β*‐globin mRNA (Faustino, Lavinha, Marini, & Moi, 1996; Myers et al., 1986). This mutation was first reported in a Portuguese individual (Faustino et al., 1992), later was discovered in a Chinese family from Guangdong Province (Jia et al., 2003), an Indian from Maharashtra (Gorakshakar et al., 2005), a Punjabi from Pakistan (Moatter et al., 2012), and a Thailand family (Prajantasen et al., 2014). The husband was also referred for the detection of thalassemia gene and found a common ‐*α* ^4.2^ deletion using gap‐PCR. When one parent was an *α*‐thalassemia carrier and the other was an *α*+*β*‐thalassemia carrier, there is a risk of having babies being homozygous for *α*‐thalassemia. Further analysis of the family revealed that the fetus inherited the rare －90 (C>T) (*HBB*: c.‐140 C>T) mutation and the common ‐‐SEA deletion from her mother, and inherited the ‐*α* ^4.2^ deletion from her father. Therefore, the fetus's phenotype was Hb H disease combined with *β*‐thalassemia. Hb H disease is the most severe non‐fatal form of α‐thalassemia, mostly caused by deletion or dysfunction of three of the four α‐globin genes. Co‐inherited *β*‐thalassemia has been described to be an improving factor of Hb H disease in a few studies (Chen, Jiang, Li, Zhou, & Li, 2018; Yin et al., 2012; Zarei, Dehbozorgian, Imanifard, Setoodegan, & Karimi, 2016). The couple was counseled that they were not at risk of having a child affected by a fatal Hb Bart's hydrops fetalis. The parents were decided chose to continue pregnancy. As expected, the infant did not showed severe anemia according follow‐up during the first 6 months of life, which verifies the reliability of molecular prenatal diagnosis.

This paper reports a rare －90 (C>T) (*HBB*: c.‐140 C>T) mutation in the Chinese population and identified a combination of －90 (C>T) (*HBB*: c.‐140 C>T) with the ‐‐^SEA^ and ‐*α* ^4.2^ deletion. Our study emphasizes that more attention is needed when common thalassemia gene mutation results are inconsistent with the results of the thalassemia screening, and accurate identification of rare mutations is important for guiding molecular detection, clinical genetic diagnosis, genetic counseling, and prenatal diagnosis for such patients.

## CONFLICT OF INTEREST

The authors have no financial conflicts of interests.

## AUTHORS’ CONTRIBUTIONS

Hou Qian prepared the majority of the manuscript including the background, case report, and discussion. Wenlan Liu designed research. Jianlin Huang and Ji Xu performed the genetic testing, and conferred the genetic diagnosis. Weihua Zhao was the original physician evaluating the patient. Xiufeng Ye reviewed the study. All authors read and approved the final manuscript.

## Data Availability

Not applicable. All data generated or analyzed during this study are included in this published article.
